# Recent fragmentation of the endangered Blakiston’s fish owl (*Bubo blakistoni*) population on Hokkaido Island, Northern Japan, Revealed by Mitochondrial DNA and Microsatellite Analyses

**DOI:** 10.1186/s40851-015-0014-3

**Published:** 2015-04-29

**Authors:** Keita Omote, Chizuko Nishida, Takeshi Takenaka, Keisuke Saito, Ryohji Shimura, Satoshi Fujimoto, Takao Sato, Ryuichi Masuda

**Affiliations:** Department of Natural History Sciences, Graduate School of Science, Hokkaido University, Sapporo, 060-0810 Japan; FILIN, Sapporo, 063-0842 Japan; Institute for Raptor Biomedicine, Kushiro, 084-0922 Japan; Kushiro City Zoo, Kushiro, 085-0201 Japan; Faculty of Letters, Keio University, Tokyo, 108-8345 Japan

**Keywords:** *Bubo blakistoni*, Genetic diversity, Microsatellite, Mitochondrial DNA haplotype, Population bottleneck, Population fragmentation

## Abstract

**Introduction:**

Blakiston’s fish owl (*Bubo blakistoni*) was previously widespread on Hokkaido Island, Japan, but is now distributed only in limited forest areas. The population size on Hokkaido decreased during the 20th century due to reduction and fragmentation of the owl’s habitat. To elucidate temporal and spatial changes in population structure and genetic diversity, we analyzed 439 individuals collected over the last 100 years.

**Results:**

We detected a population bottleneck and fragmentation event indicated by mitochondrial DNA (mtDNA) haplotype and microsatellite analyses. The lowest value for effective population size, which was estimated by moment and temporal methods from microsatellite data, occurred in the 1980s. Five haplotypes were found in the mtDNA control region; most haplotypes were previously widespread across Hokkaido, but have become fixed in separate areas after the bottleneck period. Genetic differentiation among local populations, as indicated by both mtDNA and microsatellite data, likely arose through population fragmentation.

**Conclusions:**

The owl population may have been divided into limited areas due to loss of habitats via human activities, and have lost genetic variability within the local populations through inbreeding. Our mtDNA and microsatellite data show that genetic diversity decreased in local populations, indicating the importance of individuals moving between areas for conservation of this species on Hokkaido.

## Introduction

**Figure 1:**
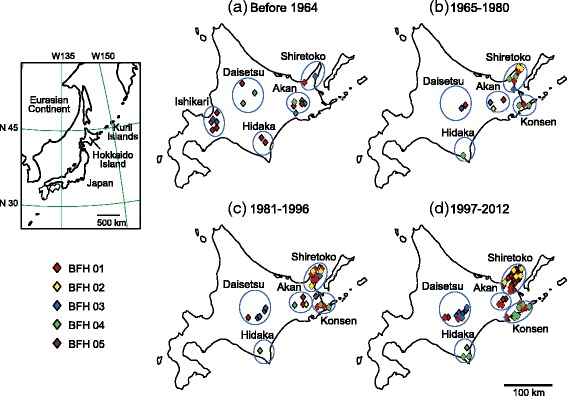
Distribution of five mtDNA control-region haplotypes through time for Blakiston’s fish owl on Hokkaido Island: **(a)** before 1964, **(b)** in intervals 1965–1980, **(c)** 1981–1996 and **(d)** 1997–2012. Color codes at the lower left indicate the haplotypes shared by individual owls. Each symbol indicates a sampling location. Circles show sampling groups analyzed as local populations. The map at the upper left shows the location of Hokkaido Island in eastern Asia.

Blakiston’s fish owl (*Bubo blakistoni*), the largest owl endemic to northeastern Asia, comprises two subspecies: *B. b. doerriesi* in continental Eurasia and *B. b. blakistoni* on Hokkaido and the southern Kuril islands (Figure 1) [1]. This species is resident in riparian forests and requires a large quantity of fish for food and large hollow trees for nesting [2,3]. It can reach ages of over 20 years and inhabits restricted territories along rivers; the clutch size is usually two eggs [3,4]. Although Blakiston’s fish owl was formerly widespread in forests on Hokkaido Island (Figure 1), its population size decreased during the 20th century due to reduction and fragmentation of its habitat through human activities. There are no records of this owl in southern Hokkaido since the 1950s, in northern Hokkaido since the 1970s, or in the Ishikari lowlands since the 1980s [5]. Today Blakiston’s fish owl survives only in eastern Hokkaido, where the population has declined since the 1970s, and the total population size on Hokkaido in the 1980s was estimated to be less than100 individuals based on field research [2,6]. For this reason, Blakiston’s fish owl has been listed as endangered on the IUCN Red List Ver.3.1, and as a National Endangered Species under Japanese law. Recent field studies have showed that, due to conservation efforts involving artificial nesting and feeding, the population has recovered to about 140 individuals in limited areas of Hokkaido. During the past 25 years, most young owls from known nest locations have been leg-banded through conservation activities by the Japanese government. Familial relationships and dispersal patterns are known for several individuals; movements of a few individuals between areas have been observed since the 1990s (T. Takenaka, personal communication).

Population genetic theory predicts that bottlenecks will lead to decreases in genetic variability, individual fitness, and the decreased capacity of populations to adapt to environmental change [7]. Fragmented populations may also face reduced gene flow, potentially leading to lower genetic diversity, inbreeding depression, fixation of deleterious mutations, and a higher risk of extinction [8]. A microsatellite analysis of the Hokkaido population of Blakiston’s fish owl (120 individuals) in the intermittent intervals (1986–1993, 1997–1999, and 2009–2010) suggested diminished genetic diversity and isolation of local populations [9]. The cause of the low level of genetic variation and gene flow was assumed to be a recent bottleneck; however, no genetic data has demonstrated the changes in population size and structure before and after the bottleneck event assumed from field observations. In the present study, to clarify the temporal changes of population structure and genetic diversity in the Hokkaido population over longer period, we used microsatellite and mitochondrial DNA (mtDNA) analyses on more current, museum, and archaeological samples collected for the last century.

## Materials and Methods

### Sampling and DNA extraction

Total DNA was extracted from blood, tissue, or cultured fibroblast samples obtained from 405 *B. blakistoni* individuals (Table 1) using the DNeasy Blood & Tissue Kit (Qiagen). Most samples came from young wild owls captured at nests for banding, and the year and location of birth of these owls were known. For the last 20 years, the samples included most owls born in Hokkaido, and most wild owls injured or killed in accidents had been banded. The blood was non-invasively collected from each owl for health check by a veterinarian as the activities to conserve the Blakiston’s fish owl by the Ministry of the Environment, Japan. A few drops of the blood were preserved in ethanol or dried on filter paper, and tissues were preserved in ethanol and frozen at −20°C until use. Fibroblasts obtained by culturing small pieces of skin tissues were frozen in liquid nitrogen until use. Total DNA was extracted from feather roots from 31 stuffed specimens (Table 1) by using the QiaAmp DNA Micro Kit (Qiagen). The stuffed specimens had been stored in museums or research institutes, with the oldest dating from the 1880s. In addition, using the method of Masuda et al. [10], total DNA was extracted from three archaeological bones from different individuals, which were buried from the late 19th century to 1939 in the Nijibetsu site in the Akan area [11]. To prevent contamination of external DNA, extraction of DNA from old samples of feathers, skin, and bones was made in clean benches on different floor from the laboratory where we used flesh samples. We used disposable pipette tips and microtubes for DNA extraction and the following experiments. These samples came from the following areas: the Ishikari Lowland, where this species is now locally extinct; the Daisetsu and Hidaka Mountains; the Akan and Konsen areas; and the Shiretoko Peninsula (Figure 1). For analyses, the samples were divided into six groups based on geographic distances and putative barriers, such as cities or farm areas. The interval used for partition data in statistical analysis was eight or 16 years; this corresponds to the average generation or the life span of the Blakiston’s fish owl.

**Table 1:** **Numbers of Blakiston’s fish owls examined, by sampling locations and periods**

**Period**	**Sampling locations**						**Total**
	**Ishikari**	**Hidaka**	**Daisetsu**	**Akan**	**Konsen**	**Shiretoko**	
pre–1964	[6]	1 [1]	[3]	[3] 3*		[2]	19
1965–1980		[1]	[2]	3	3	1 [6]	16
1981–1988		[1]	2	3 [1]	7 [1]	4 [3]	22
1989–1996		1	17	12	21 [1]	29	81
1997–2004			25	21	36	44	126
2005–2012		17	29	20	39	70	175
Total	6	22	78	66	108	159	439

Numerals without brackets show numbers of tissue samples, and those with brackets indicate numbers of feather roots from stuffed samples. Asterisks indicate archeological bone samples.

### Analysis of mtDNA sequences

From among samples, for which familial information was available, one sib from each family was selected per sampling interval for analyses of mtDNA, which is maternally inherited. Part of the first domain of mtDNA control region (590 base-pairs, bp) was amplified and sequenced from modern samples excluding sibs (*n* = 116) and museum and archaeological samples (*n* = 34). DNA was amplified by polymerase chain reaction (PCR) with primers reported by Omote et al. [12] or newly designed (Table 2). For analysis of old samples from museums, we designed new PCR primers to amplify three shorter fragments (78–148 bp) of the mtDNA control region. PCRs for the museum samples were performed in 20-μl volumes, each containing 10 μl of 2× Multiplex PCR Master Mix (Qiagen), 4.6 μl of double-distilled water, 2.0 μl of each primer solution (2 pmol/ml), 1.0 μl of DNA template, and 0.4 μl of bovine serum albumin (20 mg/ml); cycling conditions were 95°C for 15 min; 30 cycles of 94°C for 30 sec, 57°C for 3 min, and 72°C for 30 sec; and 72°C for 10 min. The PCR products were purified with the QIAquick PCR Purification Kit (Qiagen) and used as templates for nucleotide sequencing. Sequencing was performed with the BigDye Terminator v1.1 Cycle Sequencing Kit and an ABI 3130 or 3730 DNA automated sequencer (Applied Biosystems). On each of museum and archaeological samples, we performed PCR and sequencing at least three times to eliminate the potential for contamination and sequencing errors, and confirmed the authenticity of data.

**Table 2:** **PCR primers newly designed in the present study for amplification and/or sequencing for the Blakiston’s fish owl mtDNA control region**

**Primer**	**Nucleotide sequence (5′–3′)**	**Citation**
L16728	CCAAGGTCTGCGGCTTGAAAAG	Omote et al. [12]
Hcontrol-03-kb	TGAAGAGTTATGGTTTAGGTACG	Omote et al. [12]
Hcontrol-05-kb	GGGCATTAATGTCATGAAATTAG	This study
Lcontrol-06-kb	TACTAATCCATGCACTAATCCC	This study
Hcontrol-07-kb	GCCATGGATTGGAGTATTAATAG	This study
Lcontrol-08-kb	CAGTTGTACATTAAACCATCTAC	This study
Hcontrol-09-kb	GGCATGGATGTTATATCTTGGTG	This study
Lcontrol-12-kb	GTACTAATCACATACAATTCATG	This study
Lcontrol-14-M13f	*GTAAAACGACGGCCAG*CCATTAATGTGCTACGTATATAC	This study
Hcontrol-15-M13f	*GTAAAACGACGGCCAG*ATGGGATTAGTGCATGGATTAG	This study
Lcontrol-16-M13f	*GTAAAACGACGGCCAG*CCTATTCATGACAGAACGACA	This study

Italics indicate the attached sequences of M13-forward for sequencing.

Nucleotide sequences were aligned by using MEGA 5.0 software [13]. Haplotype diversity (*h*) and nucleotide diversity (π) were calculated using ARLEQUIN 3.5.1.2 software [14]. Genetic differences (*F*st) among local populations (Ishikari, Daisetu, Hidaka, Akan, Konsen and Shiretoko) on Hokkaido Island (Figure 1) were calculated by analysis of molecular variance (AMOVA) implemented, and tested with 10,000 permutations by using ARLEQUIN.

### Analysis of microsatellite genotypes

The 287 samples of blood, tissues, cultured fibroblasts, and feathers were genotyped for seven autosomal microsatellite loci. In addition, data from 120 samples (62 samples collected from 1986 to 1993; 37 from 1997 to 1999; 21 from 2009 to 2010) from Omote et al. [9] were also included, and in total, 407 individual were analyzed. We cited microsatellite markers designed for other owl species: Oe058, Oe128, and Oe129 [15,16]; 13D8 and 4E10.2 [17]; and FEPO5 and FEPO43 [18]. Forward primers were fluorescently labeled with 6-FAM, NED, PET, or VIC. PCR conditions were as previously reported by Omote et al. [9]. The molecular size of PCR products was determined on an ABI 3730 DNA automated sequencer using the GS600 LIZ size standard and GENEMAPPER 4.0 software (Applied Biosystems).

Departures from Hardy-Weinberg equilibrium (HWE) and linkage disequilibrium (LD) were tested for each microsatellite locus by using ARLEQUIN. The observed (*H*o) and expected (*H*e) heterozygosities were calculated using ARLEQUIN. Allelic richness (*A*r) and inbreeding coefficient (*F*is) were calculated with FSTAT 2.9.3 software [19]. Changes among sampling periods of *H*o, *H*e, *A*r and *F*is values were tested by pairwise non-parametric Wilcoxon signed-rank test by using STATISTICA 10 software (StatSoft). Because the sample size in older periods were small, *F*st values among local populations were calculated for each locus and combined by jackknifing, providing confidence intervals via bootstrapping, using FSTAT, and tested with 10,000 permutations by using ARLEQUIN. Effective population size (*N*e) was estimated by moment method, the bias-corrected version of the method based on LD [20], and temporal method using moment biased F-statistics [21] by using NeEstimater 2.01 software [22]. In the moment method, *N*e was estimated on each sampling period with monogamous mating model. In the temporal method, *N*e was estimated between continuous two sampling periods, assuming that generation time was five to ten years and that census size was 100 to 150. Populations that have undergone a bottleneck often exhibit a reduction in allele number and *H*o, with allele number decreasing faster than *H*o [23]. Thus, after a bottleneck, *H*o is larger than *H*e estimated from allele frequencies assuming mutation-drift equilibrium [24]. Based on this effect, known as heterozygosity excess, whether a population bottleneck had occurred was tested with BOTTLENECK 1.2.0.2 software [24,25]. To identify heterozygosity excesses, the Wilcoxon signed-rank test was used in default settings under three mutation models: infinite alleles (IAM), two-phased (TPM), and stepwise mutation (SMM).

To determine the number of genetic clusters that best fit the microsatellite data, a Bayesian assignment analysis was performed with STRUCTURE 2.3 software [26,27]. The STRUCTURE program groups multilocus data into *K* clusters without considering population origin. The STRUCTURE analysis was run for 100,000 iterations after a burn-in of 50,000 iterations, using the admixture model, and posterior probabilities were estimated for *K* = 1 through *K* = 7. For each value of *K*, 10 independent runs were conducted to quantify the amount of variation in the likelihood value for *K*. To determine the probable number of clusters, Δ*K* was calculated [28]. Individual sets of 10 replicate STRUCTURE runs were aligned using CRUMPP software [29]. Changes in *H*o for the next 50 years were simulated based on the latest genotype data in the 2005–2012 interval by using BOTTLESIM 2.6.1 software [30]. We assumed a single population without barrier against random mating (*N*e = 40) or completely isolated five local populations in equal sizes (*N*e = 8). The *N*e values were based on estimation by moment and temporal methods in the latest interval. For estimates, 1000 iterations were performed with the following constant parameters: mean life span = 16 years; age at maturity = 2 years; completely overlapping generations; each female mates with a single male each year; and sex ratio = 1:1.

## Results

### Variation and distribution of mtDNA haplotypes

Part of the mtDNA control region (590 bp) was sequenced for 150 individuals, including the old museum and archaeological samples; sequences were obtained for 31 stuffed specimens and three archeological bones. Based on five substitution sites, five haplotypes were detected in the Hokkaido Blakiston’s fish owl population and designated BFH01–BFH05 (Table 3). One of the five (BFH05) occurred in samples collected before 1964, but not in any subsequent samples. Before 1964, three haplotypes (BFH01, BFH03, and BFH04) were widespread on Hokkaido (Figure 1a). In the 1965–1980 and 1981–1996 intervals, most haplotypes occurred in several areas (Figure 1b and c), although the population of the species disappeared from the Ishikari area. By contrast, in the 1997–2012 interval, most haplotypes except for BFH01 were more restricted in distribution (Figure 1d), and the haplotype components had markedly changed. In the pre-1964 and 1965–1980 intervals, AMOVA for the haplotype data detected no genetic differences among the populations on Hokkaido (Table 4). After 1981, however, *F*st values among the populations were significantly high (Table 4). Changes of *h* and π showed similar trends (Table 4). In the overall Hokkaido population, both values slightly declined without significance; however, these in the local populations significantly decreased from pre-1964 to 1997–2012 intervals.

**Table 3:** **Polymorphic sites of the mtDNA control region haplotypes identified from the Blakiston’s fish owls**

	**Polymorphic sites**				
Haplotype	146	148	204	292	293
BFH01	T	C	C	C	C
BFH02	.	.	T	.	.
BFH03	C	T	.	.	.
BFH04	C	T	.	.	T
BFH05	C	T	.	T	.

Dots indicate nucleotides identical with those of BFH01.

**Table 4:** **Genetic differentiations and diversities caluculated by mtDNA haplotype and microsatellite data for the periods indicated**

	**mtDNA haplotype**						**Microsatellite**					
**Period**	**n**	***F*** **st**	***h*** **all**	***h*** **local**	**π** **all**	**π** **local**	**n**	***F*** **st**	***A*** **r**	***H*** **o**	***H*** **e**	***F*** **is**
pre-1964	19	0.05	0.68	0.59	0.0074	0.0066						
1965–1980	16	−0.150	0.78	0.70	0.0085	0.0075	8	−0.07	3.7	0.59	0.58	−0.02
1981–1988	16	0.31*	0.65	0.38	0.0067	0.0058	17	0.06*	3.5	0.53	0.56	0.05
1989–1996	28	0.55*	0.71	0.38	0.0070	0.0035	81	0.19*	3.3	0.52	0.55	0.05
1997–2004	31	0.49*	0.65	0.39	0.0063	0.0036	126	0.16*	3.2	0.47	0.54	0.13
2005–2012	40	0.60*	0.59	0.28	0.0065	0.0028	175	0.13*	3.3	0.51	0.53	0.05

The following parameters are given: number of samples analyzed (n), genetic differentiation among local populations (*F*st), haplotype diversity on the overall and local populations (*h* all and *h* local), nucleotide diversity on the overall and local populations (π all and π local), mean of allelic richness (*A*r), observed (*H*o) and expected heterozigosity (*H*e), and inbreeding coefficient (*F*is). Asterisks indicate statistical significance of the *F*st values (*P* < 0.05).

### Diversity and population structure indicated by microsatellite analysis

**Figure 2:**
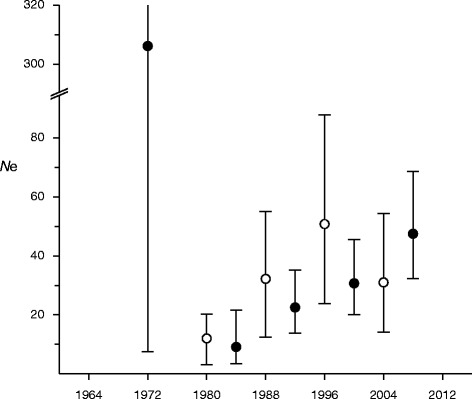
Effective population sizes (*N*e) estimated by moment method (filled circles) for each period and temporal method (open circles) between continual periods. Error bars indicate 95% confidence intervals.

Most genotypes (99.6%) for the seven microsatellite loci examined were determined from blood, frozen tissue, or fibroblast samples, for a total of 407 samples including 120 samples from Omote et al. [9]. Attempts to amplify the microsatellite loci from old museum samples were unsuccessful. When all samples from the Hokkaido population were analyzed as a single group, five and six of the seven loci significantly departed from HWE in the samples from 1981–1996 and 1997–2012, respectively. By contrast, fewer than two loci departed from HWE in each local population, excluding the Tokachi subpopulation in the 1997–2012 interval (four of the seven loci). The loci departing from HWE were different among samples from each area and period. Significant LD was detected in 0–14 pairs of markers, but the pairs were not common among samples from each area and period. Figure 2 shows estimated *N*e values by moment method on each period and temporal method assuming that the generation time was eight years and that census population size was 100. Both methods indicated that *N*e values were 20–50 in the last 20 years, *N*e was lowest in 1980s about 10 at most 20, and moment method suggested a remarkably large value in the oldest period 1965–1980.

**Figure 3:**
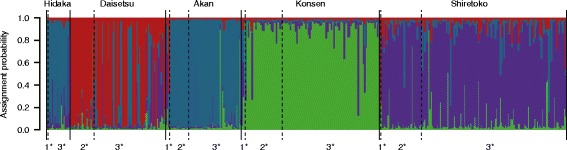
Bar plots (*K* = 4) from the STRUCTURE analysis to determine the number of genetic clusters that best fit the microsatellite data for Blakiston’s fish owl on Hokkaido. Each vertical line represents one individual, which is partitioned into four colored segments including the probability of assignment to each cluster. The individuals in each of the five local populations are arranged by sampling period: 1^*^, 1965–1980; 2^*^, 1981–1996; 3^*^, 1997–2012.

The STRUCTURE analysis showed *K* = 4 to have the highest Δ*K* value, which was more than 4× higher than Δ*K* values of the other numbers of *K*. The four clusters corresponded mostly to the local populations on Hokkaido (Figure 3). Before 1980, AMOVA for the microsatellite data detected no significant genetic differences among those local populations, but in the post-1980 intervals, *F*st values among the local populations were generally high and statistically significant (Table 4). Most individuals from the 1981–1996 interval were assigned only to clusters unique to local populations. In the pre-1980 and 1997–2012 intervals, however, some individuals were assigned to clusters predominantly representing other local populations, or not clearly assigned (Figure 3). The mean value of *A*r, which was standardized for the minimum sample size (8), was significantly decreased from the 1965–1980 interval to the 1997–2012 interval in overall Hokkaido population. Although *H*e declined slightly, *H*o decreased significantly more rapidly than *H*e from the 1981–1996 to the 1997–2012 interval (Table 4). By contrast, *H*o was often higher than *H*e in local populations after 1980, and Wilcoxon signed-rank tests showed significant heterozygosity excess under all three models (IAM, TPM, and SMM) in two of four local populations (Akan and Konsen) in the 1981–1996 interval and in two of five (Daisetsu and Akan) in the 1997–2012 interval. *F*is value was significantly increased from the pre-1980 to the 1997–2012 interval (Table 4). Simulations were used to predict changes in *H*o over the next 50 years, under two different assumptions: random mating occurs across the overall Hokkaido population, or the five local populations are completely isolated. Simulations under the respective assumptions predicted that *H*o would decrease to 0.44 and 0.28, respectively (86.5% and 56.2% of the values in the 2005–2012 interval).

## Discussion

### Temporal changes in the population structure

A previous microsatellite analysis of Blakiston’s fish owls on Hokkaido in the intermittent intervals [9] suggested that the local populations had been genetically isolated in each area. But it was not directly shown whether the local populations have been originally isolated or recently divided. To address this question, the present study analyzed a larger number of samples (442 individuals), including samples collected before the population decline, by using mtDNA haplotype and microsatellite markers.

Before 1964, most mtDNA haplotypes were distributed over large areas of Hokkaido (Figure 1a), but subsequently became restricted in distribution (Figure 1b,c and d). AMOVA for both the mtDNA haplotype and microsatellite data detected no genetic differentiation among populations in different areas before 1980 (Table 4). These results indicate that gene flow occurred across large areas of Hokkaido before 1980. Although the detailed population structure was unclear due to the smaller amount of microsatellite data before the 1980s, there may have been some genetic variation and clines between areas in historical periods. Among-population *F*st values from both the mtDNA haplotype and microsatellite data markedly increased after the 1980s (Table 4), suggesting major changes in the haplotype and allele frequencies. The separation and isolation of habitats could have rapidly grown the genetic differentiation among local populations. Departures from HWE were detected for the overall Hokkaido population in the 1981–1996 and 1997–2012 intervals; this phenomenon is known as the Wahlund effect and suggests the separation and isolation of the local populations. Because marker pairs detecting LD were not common, biased mating rather than links of loci could have caused it. Heterozygosity excess in some local populations after 1981 also indicated a recent bottleneck in local areas. The results suggest that the fragmentation of the owl population and subsequent reduction in population size have occurred recently on Hokkaido, probably around 1980 as judged from the *F*st values (Table 4). Field researches observed that the population size on Hokkaido Island was minimized in the 1970s to 1980s [2,6]. The change of *N*e estimated from microsatellite data also supported a bottleneck event around 1980s (Figure 2). Therefore, the population fragmentation occurred at the nearly the same time as the recent bottleneck. Human activities such as cultivation, housing, and building roads and dams damaged forest and river environments, and may have caused population fragmentation and bottleneck. The Blakiston’s fish owl population would have been decreased and fragmented due to loss and division of habitats via vigorous human activities around 1980s on Hokkaido.

The STRUCTURE analysis showed that the four genetic clusters mainly correspond to the local populations. Although individuals from the Hidaka and Akan areas belonged to the same cluster, the pairwise *F*st value between the two local populations was significantly higher. The Hidaka population would have been isolated rather than interacted with the Akan, and the small sample size in the Hidaka probably caused the STRUCTURE result. The 1981–1996 interval was unique in that the Daisetu, Akan, and Konsen populations were each genetically uniform. In the 1997–2012 interval, some individuals were assigned to clusters that had not previously been detected in their local populations, especially in Daisetsu (Figure 3), suggesting gene flow among local populations, and in fact a few owls were observed in the field to have moved from Akan and Shiretoko to Daisetsu and to have bred there in 1990 and 2002 (T. Takenaka, personal communication). In addition, the mtDNA haplotype diversity and microsatellite diversity have increased in Daisetsu from 1981–1996 to 1997–2012 intervals, and significant departure from HWE in Daisetsu might have been caused by the migration. Due to the small population size and long life span of Blakiston’s fish owl, these movements of a few individuals among local populations may have strongly affected the population structure and genetic diversity.

### Temporal changes in genetic diversity

Populations that undergo bottlenecks often lose genetic diversity [31]. Based on research on many threatened birds, Heber et al. [32] concluded that population bottlenecks increase hatching failure due to inbreeding and the consequent loss of genetic diversity, particularly when a population experiences a severe bottleneck to fewer than 100–150 individuals. Changes in genetic diversity have been reported in several avian species, for which samples were analyzed before and after a bottleneck. Microsatellite analysis of the endangered black-capped vireo (*Vireo atricapilla*), which apparently experienced a bottleneck early in the 20th century, showed lower genetic diversity and increased differentiation in recent (2005–2008) samples than in historical (1899–1915) samples [33]. The great prairie-chicken (*Tympanuchus cupido*) in Wisconsin, which decreased in number by 91% via loss and fragmentation of the habitat, lost genetic diversity for both mtDNA and microsatellite markers over a period of 50 years [34]. Other studies, however, have documented no changes of genetic diversity in populations that experienced bottleneck events. For example, white-tail eagle (*Haliaeetus albicilla*) populations in Europe experienced dramatic declines during the 20th century, but have retained high levels of genetic diversity, probably because of long generation time (average life span is around 17 years) [35]. The Spanish imperial eagle (*Aquila adalberti*), the population of which fragmented into small patches, exhibits reduced mtDNA *h* and π, but a microsatellite analysis showed undiminished level of nuclear diversity [36].

Blakiston’s fish owl also has long generation time, and the present study indicates that the Hokkaido population experienced bottleneck and fragmentation event. Although species with long generation times show resistance to loss of nuclear diversity, as reported by Hailer et al. [35] and Martinez et al. [36], we observed the loss of a few alleles after the recent bottleneck and detected significant decrease in *A*r and *H*o (Table 4). This indicates severe bottleneck, and *N*e value of the Hokkaido population around 1980 (Figure 2) was actually lower than *N*e values reported by Athrey et al. [33] and Johnson et al. [34] in populations during bottlenecks. Generally, bottleneck events reduce heterozygosity via loss of alleles, and make *H*e smaller than *H*o [24]. Heterozygosity excesses were observed in some local populations, indicating decline of the population size in each local area. However, *H*o was decreased more rapidly than *H*e in the overall Hokkaido population. Population fragmentation could explain the results of the present study, as it increases biased mating and cuts population into tinier local patches. The *F*is values increasing after 1981 (Table 4) indicate a higher level of inbreeding. The mean of haplotype diversity on local populations was significantly decreased much faster than haplotype diversity on the overall Hokkaido population (Table 4). Our results also support recent fragmentation of the Blakiston’s fish owl population. Both studies on white-tail eagle and Spanish imperial eagle reported undiminished levels of nuclear diversity and declines of mtDNA diversity [35,36]. Because *N*e of the mitochondrial genome is 4× lower, mtDNA haplotype diversity may be more sensitive for demographic events than nuclear diversity. The present study showed the level of genetic diversity similar to the two eagle species, but *H*o and *F*is were significantly changed. Therefore, it indicates that the Blakiston’s fish owl population on Hokkaido would have undergone more serious fragmentation and inbreeding.

Inbreeding often significantly affects birth weight, survival, reproduction, and resistance to factors such as disease, predation and environmental stress [7]. For conservation of species, we need to consider genetic factors as well as ecological factors. Our study demonstrates that the genetic diversity in local populations decreased for the last 30 years due to population fragmentation, and that even a few movements among local populations may counter this decline in genetic diversity in Blakiston’s fish owls. Simulations of changes in *H*o for the next 50 years indicated a more rapid decrease in genetic diversity when the local populations remained isolated. Therefore, conserving appropriate habitats for reproduction and promoting movements among local populations will be critical for the survival of populations and the species. Because this species has the ability to move among areas on Hokkaido, as shown in the present study, their movement should be supported and promoted through conservation projects such as corridors connecting isolated habitats.

## Conclusions

Hokkaido population of endangered Blakiston’s fish owls has been fragmented into local areas. In the present study, we clarified that most mtDNA haplotypes of old museum samples were distributed over large areas, and that the local populations have been isolated since 1980s. Microsatellite analyses and field observations showed the smallest population size on Hokkaido in around 1980s when forest and river environments were severely damaged by human activities. These indicate that the Blakiston’s fish owl population has recently decreased and fragmented due to loss and division of habitats on Hokkaido. The genetic diversity has been decreased for the last 30 years due to genetic drift and/or inbreeding in the tiny local populations. Our study demonstrates that movement of a small number of individuals between local areas could restore genetic diversity, and that promoting movements among local populations will be effective for conservation of the species. For long-term conservation management of Blakiston’s fish owls on Hokkaido, it is necessary to monitor changes in genetic diversity and study the movement of individual owls in the field.
